# Patterns and Predictors of Sentinel Lymph Node Biopsy Utilization in High- and Low-Risk Thin Melanomas

**DOI:** 10.1245/s10434-025-19079-7

**Published:** 2026-01-16

**Authors:** Mackenzie M. Mayhew, Emily G. Tocco, Margaret G. Mercante, Nidhi Kuchimanchi, Chengli Shen, Mohamad El Moheb, Samantha Ruff, Lynn T. Dengel, Craig L. Slingluff, Russell G. Witt

**Affiliations:** 1 Department of Surgery, University of Virginia, Charlottesville, VA USA; 2 University of Virginia Comprehensive Cancer Cener, Charlottesville, VA USA

**Keywords:** Cutaneous melanoma, Sentinel lymph node biopsy, Lymph node metastasis, Risk calculator, Clinical guidelines

## Abstract

**Background:**

Sentinel lymph node biopsy (SLNB) is recommended for patients with >10% risk of metastasis and not for those with <5% risk. The National Comprehensive Cancer Network acknowledges that individualized risk assessment tools, such as the Melanoma Institute of Australia (MIA) sentinel node metastasis risk model, may aid decision-making. We hypothesized that SLNB utilization would not differ substantially between the pre- and post-publication eras of the MIA model.

**Methods:**

We retrospectively reviewed National Cancer Database data from 2018 to 2022. Patients with thin melanomas (<1.0 mm Breslow depth) were classified into <5% or >10% sentinel node positivity risk groups using the MIA model. Demographic and pathologic characteristics were examined.

**Results:**

Of 58,119 patients with thin melanomas, 43,551 met inclusion criteria after excluding 14,491 with intermediate (5–10%) risk. Among 3949 with >10% risk, 62.9% had SLNB and 37.1% had SLNB omitted. Conversely, among 39,602 with <5% risk, 13.7% received SLNB. Tumor thickness, ulceration, and mitotic rate were significant predictors of SLNB in both groups. Increasing age was associated with lower odds of SLNB only in the low-risk cohort. Notably, SLNB utilization changed minimally following publication of the MIA model, increasing by <1% in the <5% group and 4% in the >10% group.

**Conclusions:**

Before and after MIA model publication, SLNB was underutilized in high-risk patients and overutilized in low-risk patients. Utilization was primarily driven by tumor-specific factors. Our findings suggest limited early adoption of the model and highlight the need for improved dissemination of personalized risk stratification tools.

**Supplementary Information:**

The online version contains supplementary material available at 10.1245/s10434-025-19079-7.

In the management of melanoma, several risk factors have been identified to predict the likelihood of a positive sentinel lymph node biopsy (SLNB), including mitotic index, lymphovascular invasion (LVI), tumor thickness, and age.^1^ Current National Comprehensive Cancer Network guidelines base recommendations for SLNB primarily on tumor thickness and pathologic features^2^, while acknowledging that individualized risk estimation may support shared decision-making. In general, SLNB is routinely recommended for those with an estimated sentinel node positivity risk >10%, may be considered selectively for those with intermediate risk, and is not routinely recommended when the estimated risk is <5%.

Although most thin melanomas are associated with a risk of metastasis <5%, certain pathological and patient characteristics may elevate this risk, creating uncertainty in clinical decision-making. In this context, validated risk prediction tools, such as those developed by the Melanoma Institute of Australia (MIA) and Memorial Sloan Kettering Cancer Center (MSKCC), are available to estimate the likelihood of sentinel node involvement and may serve as adjuncts to clinicopathologic assessment and patient-centered discussion.^3^

The MIA developed the Sentinel Node Metastasis Risk Prediction Tool (MIA model), which was made publicly available in June 2020 through launching an online publicly accessible risk calculator.^4^ The Sentinel Node Metastasis Risk Prediction Tool was created based on the MIA database, and it was later externally validated by the University of Texas MD Anderson Cancer Center.^5^ The MIA model provides a risk score based on patient age, histology, mitotic rate, LVI, ulceration, and tumor depth. As the most recently developed and externally validated SLN risk prediction model available, the MIA model offers an updated framework for estimating nodal risk and was therefore used in this study to stratify patients by predicted risk.

The purpose of this study was to evaluate national patterns of SLNB utilization in patients with thin melanoma (<1.0 mm) and to determine whether practice meaningfully changed following publication of the MIA model in 2020. Specifically, we examined the frequency with which high-risk patients (>10% predicted risk) did not undergo SLNB and low-risk patients (<5% risk) received it, both before and after model publication. We hypothesized that substantial discordance would persist during both periods, indicating limited early adoption of individualized risk-prediction tools. By defining current utilization patterns, this study aims to assess whether the MIA model is influencing clinical decision-making and to identify opportunities for improved dissemination and education regarding individualized SLNB risk stratification.

## Methods

This retrospective cohort study analyzed data from the National Cancer Database (NCDB) collected between 2018 and 2022. The NCDB, a joint project of the Commission on Cancer of the American College of Surgeons and the American Cancer Society, captures cancer cases in the United States from commission-accredited cancer programs.^6^ The data used in this study were derived from de-identified NCDB files. The American Cancer Society and Commission on Cancer have not verified the data and are not responsible for the analytic or statistical methodology employed or the conclusions drawn in this report. This study was exempt from informed consent requirements by the University of Virginia institutional review board, as it used publicly available dataset with de-identified information.

### Study Groups and Variables

Patients with melanoma <1.0 mm in Breslow depth were identified from the NCDB. Study variables included demographic factors such as age, race, ethnicity, Charlson–Deyo comorbidity index, insurance status, and type of treatment facility. Melanoma-specific factors included histology, primary tumor site, Breslow depth, mitotic rate, ulceration, and LVI.

**Fig. 1:**
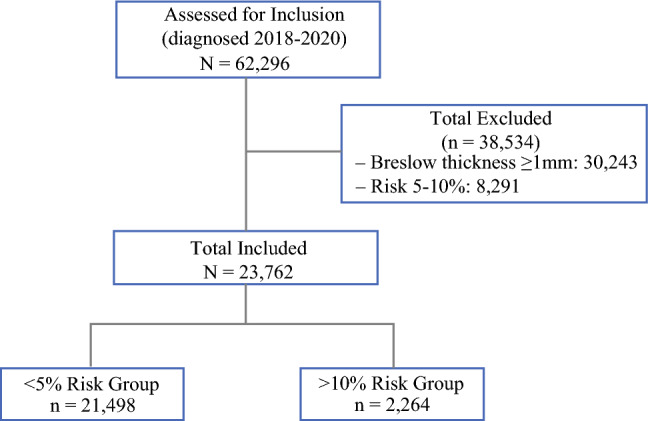
Flowchart outlining inclusion and exclusion criteria for study cohort.

Risk groups were classified using the MIA model.^4^ Patients were stratified into low-risk (<5%) and high-risk (>10%) groups. Those with an intermediate risk (5–10%) were excluded from the analysis because there are no definitive recommendations for SLNB usage in this group (Figure 1).

### Primary Outcomes

The primary outcomes of the study included the percentage of patients in each risk group categorized by demographic and melanoma-specific characteristics, the proportion of SLNB procedures performed within each risk group prior to and after the dissemination of the MIA model, the demographic characteristics of patients undergoing SLNB, and the independent predictors of SLNB use in both low- and high-risk groups.

### Statistical Analysis

Descriptive statistics were used to summarize patient demographic characteristics, tumor-specific factors, and facility characteristics for the overall cohort and stratified by SLNB status within each risk group (<5% and >10% predicted risk), as defined by the MIA model. Categorical variables were reported as frequencies and percentages, and continuous variables were summarized using medians and interquartile ranges.

Univariable logistic regression was performed to evaluate associations between patient demographics, tumor-specific factors, and facility characteristics with SLNB utilization in both the high-risk (>10%) and low-risk (<5%) groups. Covariates with a p-value <0.05 in univariable analysis were included in multivariable logistic regression models to identify independent predictors of SLNB use. Adjusted odds ratios (aORs) with corresponding 95% confidence intervals (CIs) were reported for significant predictors in the multivariable model. To evaluate changes in SLNB utilization before and after publication of the MIA model, we conducted an interrupted time series analysis. Overall survival in the high-risk group was evaluated using Kaplan–Meier methods and compared with the log-rank test, followed by multivariable Cox proportional hazards modeling to estimate adjusted hazard ratios (HRs). All analyses were conducted using R Studio (version 4.4.1). A two-tailed p-value <0.05 was considered statistically significant.

## Results

### General Demographics

Of the 58,119 patients with melanoma <1.0 mm in the NCDB dataset, 14,491 were excluded for falling between <5% and >10% risk and 77 were excluded because a risk score could not be calculated, leaving 43,551 patients included in our study. As expected, based on the MIA model, patients in the high-risk group were younger and exhibited higher rates of pathological high-risk characteristics, including LVI, greater depth, higher mitotic rates, and more aggressive histology (Table 1). Within the high-risk group, there was a disproportionate number of female patients, with women comprising almost 61% of high-risk patients but only 39% of low-risk patients.

**Table 1:** Demographics and tumor characteristics by risk group

Characteristics	<5% risk group (n=39,602)	>10% risk group (n=3949)	p-Value
Patient characteristics			
Age	68 (60–76)	41 (34–49)	<0.001
Sex			<0.001
Male	24,052 (60.7)	1543 (39.1)	
Female	15,550 (39.3)	2406 (60.9)	
Race			<0.001
American Indian, Aleutian, or Eskimo	48 (0.1)	5 (0.1)	
Asian or Pacific Islander	64 (0.2)	21 (0.5)	
Black	39 (0.1)	23 (0.6)	
White	39,009 (98.5)	3828 (96.9)	
Other	118 (0.3)	24 (0.6)	
Unknown	324 (0.8)	48 (1.2)	
Ethnicity			<0.001
Hispanic	310 (0.8)	80 (2.1)	
Non-Hispanic	38,626 (99.2)	3808 (97.9)	
Charlson–Deyo Comorbidity Index			<0.001
0	32,788 (82.7)	3634 (92.0)	
1	4274 (10.8)	243 (6.2)	
2	1463 (3.7)	34 (0.9)	
3	1127 (2.8)	38 (1.0)	
Treatment facility type			<0.001
Academic/research program	21,335 (53.9)	1188 (30.1)	
Community Cancer Program	1432 (3.6)	82 (2.1)	
Comprehensive Community Cancer Program	9273 (23.4)	522 (13.2)	
Integrated Network Cancer Program	7481 (18.9)	439 (11.1)	
Not available	81 (0.2)	1718 (43.5)	
Insurance			<0.001
Medicaid	771 (1.9)	272 (6.9)	
Medicare	22,475 (56.8)	281 (7.1)	
Other Government	549 (1.4)	75 (1.9)	
Private insurance	15,184 (38.3)	3226 (81.7)	
Uninsured	279 (0.7)	70 (1.8)	
Unknown	344 (0.9)	25 (0.6)	
Tumor characteristics			
Site			<0.001
Head/neck	9930 (25.1)	570 (14.4)	
Trunk	13,394 (33.8)	1262 (32.0)	
Upper extremity	10,605 (26.8)	972 (24.6)	
Lower extremity	5557 (14.0)	1130 (28.6)	
Other	34 (0.1)	4 (0.1)	
Unavailable	82 (0.2)	11 (0.3)	
Histology			<0.001
Acral lentiginous	116 (0.3)	304 (7.7)	
Desmoplastic	296 (0.7)	0 (0.0)	
Lentigo maligna	9354 (23.6)	8 (0.2)	
Nodular	816 (2.1)	134 (3.4)	
Superficial spreading	29,020 (73.3)	3503 (88.7)	
Lymphovascular invasion			<0.001
Absent	39,587 (100.0)	3596 (91.1)	
Present	15 (0.0)	353 (8.9)	
Ulceration			<0.001
Absent	38,958 (98.4)	3471 (87.9)	
Present	644 (1.6)	478 (12.1)	
Breslow thickness			<0.001
<0.8 mm	34,862 (88.0)	1905 (48.2)	
≥0.8 mm	4740 (12.0)	2044 (51.8)	
Mitotic rate			<0.001
0	35,968 (90.8)	453 (11.5)	
1	2519 (6.4)	1926 (48.8)	
≥2	1115 (2.8)	1570 (39.8)	

Data are presented as median (interquartile range) or n (%) unless otherwise indicated.

SLNB, sentinel lymph node biopsy

The proportion of patients undergoing SLNB increased with risk category, with 13.7% of low-risk patients and 62.9% of high-risk patients receiving the procedure (Table 2). Of those who underwent SLNB, the percent with positive SLNB was consistent with the MIA model estimates, with 4.2% positive in the low-risk group and 11.6% in the high-risk group.

**Table 2:** Sentinel lymph node biopsy status and results by risk group

Biopsy status/results	<5% risk group (n=39,602)	>10% risk group (n=3949)
SLNB		
Received	5433 (13.7)	2485 (62.9)
Not received	34,156 (86.2)	1463 (37.1)
Unknown	13 (0.1)	1 (0.0)
Sentinel node status^a^		
Positive	229 (4.2)	289 (11.6)
Negative	5202 (95.7)	2196 (88.4)
Unknown	2 (0.1)	0 (0.0)

Data are presented as n (%).

SLNB, sentinel lymph node biopsy.

^a^Includes only those who received SLNB

### Low-Risk Melanoma

In the low-risk group, sex, Charlson–Deyo comorbidity index, treatment facility type, histology, depth, ulceration, and mitotic rate were significantly different between those who received SLNB and those who did not on univariate analysis (Table 3). Although SLNB utilization differed significantly by year of diagnosis (2018–2020: 13.4% vs 2021–2022: 14.1%; p=0.043), the absolute change was small, amounting to less than a 1% increase. Breslow thickness ≥0.8 mm, younger age, presence of ulceration, higher mitotic rate (1/mm^2^ and ≥2/mm^2^), and treatment at an integrated network cancer program or comprehensive community cancer center were all independent predictors of SLNB for patients with a <5% risk (Table 4). Patients diagnosed in 2021–2022 had a slightly higher likelihood of undergoing SLNB than those diagnosed in 2018–2020 (aOR 1.08, p=0.036).

**Table 3:** Demographics and tumor characteristics of patients in the <5% risk group stratified by biopsy status

Characteristics	No SLNB (n=34,156)	Received SLNB (n=5433)	p-Value
Patient characteristics			
Age	68 (60–76)	68 (61–75)	0.138
Sex			<0.001
Male	20,511 (60.1)	3534 (65.0)	
Female	13,645 (39.9)	1899 (35.0)	
Race			0.042
American Indian, Aleutian, or Eskimo	46 (0.1)	10 (0.2)	
Asian or Pacific Islander	30 (0.1)	11 (0.2)	
Black	31 (0.1)	8 (0.1)	
White	33,642 (98.5)	5354 (98.5)	
Other	13 (0.0)	2 (0.0)	
Unknown	394 (1.2)	48 (0.9)	
Ethnicity			0.104
Hispanic	260 (0.8)	50 (0.9)	
Non-Hispanic	33,306 (97.5)	5307 (97.7)	
Unknown	590 (1.7)	76 (1.4)	
Charlson–Deyo Comorbidity Index			<0.001
0	28,393 (83.1)	4336 (79.8)	
1	3579 (10.5)	693 (12.8)	
2	1215 (3.6)	247 (2.5)	
3	969 (2.8)	157 (2.9)	
Year of diagnosis			0.043
2018–2020	19,307 (86.8)	2991 (13.4)	
2021–2022	14,849 (85.9)	2442 (14.1)	
Treatment facility type			<0.001
Academic/research program	18,927 (55.4)	2403 (44.2)	
Community Cancer Program	1265 (3.7)	165 (3.0)	
Comprehensive Community Cancer Program	7899 (23.1)	1368 (25.2)	
Integrated Network Cancer Program	6004 (17.6)	1477 (27.2)	
Not available	61 (0.2)	20 (0.4)	
Insurance			<0.001
Medicaid	672 (2.0)	98 (1.8)	
Medicare	19,293 (56.5)	3176 (58.5)	
Other Government	451 (1.3)	98 (1.8)	
Private insurance	13,219 (38.7)	1960 (36.1)	
Uninsured	239 (0.7)	40 (0.7)	
Unknown	282 (0.8)	61 (1.1)	
Tumor characteristics			
Site			0.124
Head/neck	8530 (25.0)	1397 (25.7)	
Trunk	11,590 (33.9)	1800 (33.1)	
Upper extremity	9110 (26.7)	1489 (27.4)	
Lower extremity	4830 (14.1)	727 (13.4)	
Other	31 (0.1)	3 (0.1)	
Histology			<0.001
Acral lentiginous	88 (0.3)	28 (0.5)	
Desmoplastic	154 (0.5)	142 (2.6)	
Lentigo maligna	8284 (24.3)	1065 (19.6)	
Nodular	386 (1.1)	430 (7.9)	
Superficial spreading	25,244 (73.9)	3768 (69.4)	
Lymphovascular invasion			0.967
Absent	34,143 (100.0)	5431 (100.0)	
Present	13 (0.0)	2 (0.0)	
Ulceration			<0.001
Absent	33,829 (99.0)	5117 (94.2)	
Present	327 (1.0)	316 (5.8)	
Breslow thickness			<0.001
<0.8 mm	32,271 (94.5)	2579 (47.5)	
≥0.8 mm	1885 (5.5)	2854 (52.5)	
Mitotic rate			<0.001
0	31,712 (92.8)	4245 (78.1)	
1	1832 (5.4)	686 (12.6)	
≥2	612 (1.8)	502 (9.2)	

Data are presented as median (interquartile range) or n (%) unless otherwise indicated.

SLNB, sentinel lymph node biopsy.

**Table 4:** Independent predictors of sentinel lymph node biopsy in the <5% risk group

Variable	aOR (95% CI)	p-Value
Age	0.97 (0.96–0.97)	<0.001
Facility type (ref: community program)		
Comprehensive Community Cancer Program	1.30 (1.07–1.60)	0.009
Integrated Network Cancer Program	2.14 (1.76–2.63)	<0.001
Insurance		
Medicare	1.25 (1.13–1.36)	<0.001
Other Government	1.37 (1.04–1.78)	0.023
Breslow thickness (ref: <0.8mm)		
≥0.8 mm	19.4 (17.9–20.9)	<0.001
Ulceration (ref: absent)		
Present	5.60 (4.60–6.81)	<0.001
Lymphovascular invasion (ref: absent)		
Present	0.80 (0.08–5.50)	0.844
Mitotic rate (ref: 0)		
1	1.95 (1.72–2.21)	<0.001
≥2	3.13 (2.63–3.72)	<0.001
Histology (ref: superficial spreading)		
Nodular	2.52 (2.08–3.07)	<0.001
Lentigo maligna	0.81 (0.74–0.88)	<0.001
Acral lentiginous	5.05 (3.10–7.96)	<0.001
Desmoplastic	2.14 (1.57–2.91)	<0.001
Year of diagnosis (ref: 2018–2020)		
2021–2022	1.08 (1.00–1.15)	0.036

aOR, adjusted odds ratio; CI, confidence interval; ref, reference

Of note, 48% of patients with malignant desmoplastic melanoma in the low-risk group underwent SLNB. Based on the nature of the NCDB data, it is not possible to assess whether these represented purely desmoplastic melanoma or mixed desmoplastic specimens.

### High-Risk Melanoma

For the high-risk group, age, sex, treatment facility type, histology, presence of LVI, depth, ulceration, and mitotic rate were significantly different on univariate analysis between those who received SLNB and those who did not (Table 5). SLNB utilization also varied modestly by year of diagnosis in the high-risk group (2018–2020: 61.3% vs 2021–2022: 65.3%; p=0.013). Independent predictors of SLNB for patients with more than a 10% risk were treatment at an integrated network cancer program, Breslow thickness ≥0.8 mm, presence of ulceration, mitotic rates of 1/mm^2^ and ≥2/mm^2^ (Table 6). While year of diagnosis appeared to be associated with SLNB use in unadjusted comparisons, this effect did not persist after adjustment for clinical and demographic factors (aOR 1.06, p=.429). Unadjusted Kaplan–Meier analysis showed no significant survival difference between those who underwent SLNB and those who did not. However, after adjustment for age, comorbidity, tumor characteristics, and facility type in a multivariable Cox model, SLNB was associated with a significant reduction in mortality (HR 0.91; 95% CI 0.84–0.99, p=0.041).

**Table 5:** Demographics and tumor characteristics of patients in the >10% risk group stratified by biopsy status

Characteristics	No SLNB (n =1463)	Received SLNB (n =2485)	p-Value
Patient characteristics			
Age	39 (33–47)	43 (35–50)	<0.001
Sex			0.004
Male	529 (36.2)	1013 (40.8)	
Female	934 (63.8)	1472 (59.2)	
Race			0.102
American Indian, Aleutian, or Eskimo	7 (0.5)	3 (0.1)	
Asian or Pacific Islander	1 (0.1)	5 (0.2)	
Black	11 (0.8)	12 (0.5)	
White	1413 (96.6)	2414 (97.1)	
Other	6 (0.4)	4 (0.2)	
Unknown	25 (1.7)	47 (1.9)	
Ethnicity			0.572
Hispanic	26 (1.8)	54 (2.2)	
Non-Hispanic	1412 (96.5)	2395 (96.4)	
Unknown	25 (1.7)	36 (1.4)	
Charlson–Deyo Comorbidity Index			0.204
0	1353 (92.5)	2280 (91.8)	
1	78 (5.3)	165 (6.6)	
2	14 (1.0)	20 (0.8)	
3	18 (1.2)	20 (0.8)	
Year of diagnosis			0.013
2018–2020	896 (38.7)	1420 (61.3)	
2021–2022	567 (34.7)	1065 (65.3)	
Treatment facility type			<0.001
Academic/Research Program	393 (26.9)	794 (32.0)	
Community Cancer Program	30 (2.1)	52 (2.1)	
Comprehensive Community Cancer Program	192 (13.1)	330 (13.3)	
Integrated Network Cancer Program	110 (7.5)	329 (13.2)	
Not available	738 (50.4)	980 (39.4)	
Insurance			<0.001
Medicaid	112 (7.7)	160 (6.4)	
Medicare	121 (8.3)	160 (6.4)	
Other Government	15 (1.0)	60 (2.4)	
Private insurance	1171 (80.0)	2054 (82.7)	
Uninsured	34 (2.3)	36 (1.4)	
Unknown	10 (0.7)	15 (0.6)	
Tumor characteristics			
Site			0.443
Head/neck	212 (14.5)	358 (14.4)	
Trunk	482 (32.9)	780 (31.4)	
Upper extremity	341 (23.3)	630 (25.4)	
Lower extremity	425 (29.0)	705 (28.4)	
Other	0 (0.0)	4 (0.2)	
Unavailable	3 (0.2)	8 (0.3)	
Histology			<0.001
Acral lentiginous	139 (9.5)	165 (6.6)	
Desmoplastic	0 (0.0)	0 (0.0)	
Lentigo maligna	3 (0.2)	5 (0.2)	
Nodular	32 (2.2)	102 (4.1)	
Superficial spreading	1289 (88.1)	2213 (89.1)	
Lymphovascular invasion			<0.001
Absent	1298 (88.7)	2298 (92.5)	
Present	165 (11.3)	187 (7.5)	
Ulceration			<0.001
Absent	1367 (93.4)	2103 (84.6)	
Present	96 (6.6)	382 (15.4)	
Breslow thickness			<0.001
<0.8 mm	1174 (80.2)	730 (29.4)	
≥0.8 mm	289 (19.8)	1755 (70.6)	
Mitotic rate			<0.001
0	253 (17.3)	199 (8.0)	
1	784 (53.6)	1142 (46.0)	
≥2	426 (29.1)	1144 (46.0)	

Data are presented as median (interquartile range) or n (%) unless otherwise indicated.

SLNB, sentinel lymph node biopsy.

**Table 6:** Independent predictors of sentinel lymph node biopsy in the >10% risk group

Variable	aOR (95% CI)	p-Value
Age	0.99 (0.98–1.01)	0.267
Sex (ref: male)	0.99 (0.84–1.16)	0.862
Facility type (ref: community program)		
Integrated Network Cancer Program	1.94 (1.06–3.51)	0.030
Insurance (ref: private)		
Medicare	0.54 (0.38–0.78)	0.001
Other Government	2.39 (1.28–4.65)	0.007
Uninsured	0.39 (0.22–0.68)	<0.001
Breslow thickness (ref: <0.8 mm)		
≥0.8 mm	9.96 (8.46–11.8)	<0.001
Ulceration (ref: absent)		
Present	2.73 (2.07–3.64)	<0.001
Lymphovascular invasion (ref: absent)		
Present	1.12 (0.77–1.62)	0.564
Mitotic rate (ref: 0)		
1	1.49 (1.12–1.99)	0.007
≥2	2.49 (1.83–3.42)	<0.001
Year of diagnosis (ref: 2018–2020)		
2021–2022	1.06 (0.91–1.24)	0.429

aOR, adjusted odds ratio; CI, confidence interval.

Notably, histologic subtype was an independent predictor of SLNB use in the low-risk group but not in the high-risk group. Among patients in the low-risk group, nodular, acral lentiginous, and desmoplastic melanomas were all associated with significantly higher odds of SLNB than was superficial spreading melanoma, whereas lentigo maligna was associated with lower odds. Insurance status also demonstrated divergent effects across risk groups. In the low-risk group, Medicare was associated with increased odds of SLNB, while in the high-risk group Medicare coverage and uninsured status were associated with decreased SLNB utilization. Tumor characteristics, including Breslow thickness, ulceration, and mitotic rate, influenced SLNB prediction across both groups, whereas LVI had no impact.

Interrupted time series analysis demonstrated no measurable change in SLNB utilization following publication of the MIA model (**Supplementary Figure 1**). In the <5% risk cohort, there was no significant pre-intervention trend in SLNB use (OR 1.00; 95% CI 0.92–1.10), no immediate change after the model became available (OR 0.95; 95% CI 0.81–1.10), and no difference between pre- and post-publication slopes (OR 1.06; 95% CI 0.94–1.20). Similarly, in the >10% risk group, none of the pre-intervention trend (OR 0.91, p=0.37), the immediate change (OR 1.07, p=0.69), or the post-intervention slope (OR 1.20, p=0.21) reached significance.

## Discussion

This study examined the utilization of SLNB in patients with thin melanomas (<1.0 mm Breslow depth) before and after the widespread availability of the MIA model. Among high-risk patients, a total of 62.9% underwent SLNB and 37.1% did not, despite their elevated predicted risk. Although SLNB use in this group increased modestly by 4.0% after 2020, year of diagnosis was not an independent predictor of SLNB receipt, indicating minimal change in practice following publication of the MIA model. Conversely, overall SLNB utilization in the low-risk group was 13.7%, with a slight increase of 0.7% following 2020 despite their low risk. Among those who underwent the procedure, the MIA model accurately predicted the rates of sentinel lymph node metastasis. Predictors of SLNB utilization included tumor-specific factors such as Breslow depth, ulceration, and mitotic rate, as well as facility type and age for the low-risk group. Taken together, these findings suggest that, both before and after the introduction of the MIA model, SLNB was primarily performed based on traditional pathologic risk factors. However, more than one-third of high-risk patients still did not undergo SLNB, reflecting persistent underuse that may stem from under-recognition of high-risk features or from factors not captured in the NCDB, including patient or physician preference, referral patterns, resource availability, or cost. Notably, SLNB practice patterns did not substantially change after publication of the MIA model, demonstrating limited real-world adoption of the tool in the years examined.

Although the SLNB rate for thin melanomas is relatively low overall, certain known factors confer a higher risk of SLN metastasis. These risk factors include younger age, LVI, higher mitotic rate, and head/neck tumor location.^1^ Our findings indicate that age is an independent predictor of having an SLNB performed in the low-risk group, and mitotic rate serves as an independent predictor in both groups. These findings suggest that surgeons consider well-recognized pathological risk factors when deciding on SLNB. Interestingly, LVI was not an independent predictor of SLNB use in our cohort, despite the known association with increased lymph node metastasis. Histologic subtype also influenced SLNB decision-making in the low-risk group, but not in the high-risk group. Nodular, acral lentiginous, and desmoplastic melanomas were all associated with significantly higher odds of undergoing SLNB compared with superficial spreading melanoma, whereas lentigo maligna was associated with lower SLNB utilization. This pattern is notable because pure desmoplastic melanomas typically carry a lower risk of sentinel node metastasis.^2,7^ However, we were unable to distinguish between pure and mixed subtypes within the NCDB, which may account for some of this effect. Although histologic subtype is not traditionally emphasized as an independent prognostic factor by the National Comprehensive Cancer Network, it is incorporated into the MIA model, and our findings suggest that histology may inadvertently influence clinician–patient discussions and contribute to SLNB overuse in patients whose predicted risk remains below guideline thresholds. The association between younger age and increased SLNB utilization does not appear to be driven by comorbidities in older patients, as a higher Charlson–Deyo Comorbidity Index was not significantly associated with SLNB omission. With regard to the high-risk group, it is unclear what unmeasured factors influenced SLNB omission, but it may be reflective of under-recognition of risk factors such as age.

Receiving treatment at an integrated network cancer program (INCP), defined as a group of facilities offering integrated, comprehensive cancer care under centralized governance, was an independent predictor for receiving SLNB in both groups, and treatment at a comprehensive community cancer program independently predicted SLNB use in the low-risk cohort. Treatment at INCPs, as well as at academic and non-academic comprehensive community cancer programs have been associated with better overall patient survival rates for head and neck cancers than treatment at community cancer programs.^8^ Despite this, our analysis found that INCPs and comprehensive community cancer programs were independent predictors of performing SLNB in patients with low risk of sentinel lymph node metastasis, suggesting a potential pattern of overutilization within higher-resource, more specialized centers. However, results for the high-risk group should be interpreted with caution because treatment facility type was unavailable for a substantial proportion of patients, which may bias estimates of institutional practice patterns. Insurance status similarly influenced SLNB decision-making. In the low-risk group, Medicare beneficiaries were more likely to undergo SLNB, whereas in the high-risk group, Medicare coverage was associated with significantly lower odds of receiving SLNB. This paradox may relate to several interacting factors, including surgeon practice variation and the growing availability of gene expression profile (GEP) testing. Medicare reimbursement for GEP assays during this time may have led to more intensive staging discussions, even in the low-risk group.^9^

Important clinical implications arise from our findings. Many patients (10% in the high-risk group) anticipated to have SLN metastasis may not have adequate staging or treatment, appropriate adjuvant care, or appropriate surveillance. Our results demonstrated that SLNB was associated with a significant reduction in mortality in the high-risk group, suggesting a potential therapeutic benefit, as other literature has suggested, and reinforcing the notion that appropriately staged patients are more likely to receive appropriate adjuvant therapy and surveillance.^10^ However, this must be interpreted with the knowledge that there is limited follow-up time for some of the patients. Conversely, there is a substantial cost burden for patients receiving SLNB in the <5% risk group. The cost of SLNB for patients with thin melanomas (defined as <1.2 mm in a study analyzing the cost of treatment for thin melanomas) ranged from $US10,096 to $US15,223 compared with $US1000 to $US1740 for outpatient wide local excision alone.^11^ This leaves a $US10,000 cost burden for patients who are in the lowest risk category. Based on a study utilizing hospital cost data on SLNB in melanoma with high-risk features (defined as positive deep margins, LVI, or mitotic index ≥2/mm^2^), patients who underwent SLNB had higher median total hospital costs and higher costs across all subcomponents and nearly double the operative time.^11^ The general out-of-pocket cost for patients with Medicare was estimated to be $US652 for wide local excision followed by SLNB compared with $US79 for wide local excision alone in an outpatient clinic.^12^ This is likely more costly for patients with private insurance.^12^ Further, it costs the hospital approximately $US50,000 to identify a positive SLN (based on an SLN positivity of 6.1%) in a cohort of patients undergoing SLNB for higher-risk thin melanoma.^13^

We utilized the MIA model to stratify risk groups within the NCDB because it is well-validated, widely available, and the most recently developed prediction model in melanoma. Before the MIA model was published, the MSKCC nomogram was the most widely utilized; however, the MIA model incorporates additional predictors (LVI, mitotic rate, and histologic subtype) and excludes Clark level.^5,14,15^ The MIA model found a 9.2% increase in accuracy compared with the MSKCC nomogram and included over three times the number of patients in its analysis.^5^ Despite the evidence for improved predictive accuracy of the MIA model, our interrupted time series analysis showed no detectable change in national SLNB practice patterns after publication of the model. Although univariate differences in SLNB use were observed and persisted in adjusted analyses only for the low-risk cohort, the absolute changes were small. These trends suggest that the MIA model has not meaningfully influenced real-world clinical decision-making in the two years following its publication. Limited uptake may reflect entrenched practice patterns or the growing clinical interest in GEP tests, which some clinicians may have been using adjunctively or in place of validated risk calculators during this time period despite ongoing debate regarding their appropriate role.^2,9,16^ Additionally, some studies have found that the MIA model underestimates the risk of nodal metastasis in low-risk patients, which may be part of the decision-making process for clinicians during discussions with patients.^3,15,17^ Our findings indicate that, although the MIA model offers improved risk stratification, its availability alone has not been sufficient to shift SLNB utilization nationwide.

Our study has several limitations inherent to the use of the NCDB. Although the database provides broad national representation, it lacks important clinical granularity that may influence SLNB decision-making. The NCDB does not capture biopsy technique, presence of positive deep margin, or whether residual melanoma was present before wide local excision, all of which may play a role in clinical decision-making, particularly when biopsies raise uncertainty about true tumor depth. This limitation may contribute to discordance between predicted risk and observed SLNB utilization. Wide local excisions performed by dermatologists in office settings are not captured in this database, suggesting that we may be overestimating appropriate SLNB utilization in high-risk thin melanoma cases. Additionally, we do not have data on long-term recurrence outcomes or whether the omission of SLNB ultimately affected recurrence-free survival. The NCDB also lacks granularity in clinical details such as reasons for SLNB omission, physician decision-making processes, or patient preferences in decisions. The absence of Clark scores within the NCDB dataset precluded the use of the MSKCC melanoma sentinel lymph node metastasis nomogram for additional analysis.

## Conclusion

In this national analysis of thin melanoma, substantial discordance persisted between predicted risk and SLNB utilization, with underuse in high-risk patients and overuse in low-risk patients. Practice patterns remained unchanged following publication of the MIA model, suggesting limited early adoption. Better alignment of SLNB decisions with individualized risk estimates could reduce unnecessary procedures, optimize resource use, and ensure timely intervention for patients at highest risk. Ongoing longitudinal assessment will be needed to evaluate future uptake of risk-prediction tools and to determine whether targeted educational initiatives are warranted to support their adoption.

## Supplementary Information

Below is the link to the electronic supplementary material.

Supplementary file1 (DOCX 100 KB)

## References

[1] Shannon AB, Sharon CE, Straker RJ 3rd, et al. Sentinel lymph node biopsy in patients with T1a cutaneous malignant melanoma: A multicenter cohort study. *J Am Acad Dermatol*. 2023;88(1):52–9. 36184008 10.1016/j.jaad.2022.09.040

[2] Susan M Swetter, Douglas Johnson, Mark Albertini, et al. NCCN Guidelines Version 2.2025 Melanoma: Cutaneous.

[3] Maddineni S, Dizon MP, Muralidharan V, et al. Validation of the Melanoma Institute of Australia’s sentinel lymph node biopsy risk Prediction Tool for cutaneous melanoma. *Ann Surg Oncol*. 2024;31(4):2737–46. 38216800 10.1245/s10434-023-14862-w

[4] Melanoma Institute Australia. Melanoma risk assessment tool. https://www.melanomarisk.org.au/SNLLand. Accessed December 7, 2025.

[5] Lo SN, Ma J, Scolyer RA, et al. Improved risk prediction calculator for sentinel node positivity in patients with melanoma: The Melanoma Institute Australia nomogram. *J Clin Oncol*. 2020;38(24):2719–27. 32530761 10.1200/JCO.19.02362 PMC7430218

[6] National Cancer database. ACS. https://www.facs.org/quality-programs/cancer-programs/national-cancer-database/. Accessed December 7, 2025.

[7] Hodson M, Feustel P, Davis L. Sentinel lymph node biopsy in desmoplastic melanoma - the percent desmoplastic component matters: A systematic review. *J Plast Reconstr Aesthet Surg*. 2022;75(12):4441–9. 36283925 10.1016/j.bjps.2022.08.044

[8] Carey RM, Fathy R, Shah RR, et al. Association of type of treatment facility with overall survival after a diagnosis of head and neck cancer. *JAMA Netw Open*. 2020;3(1):e1919697. 31977060 10.1001/jamanetworkopen.2019.19697 PMC6991286

[9] Grossman D, Okwundu N, Bartlett EK, et al. Prognostic gene expression profiling in cutaneous melanoma: Identifying the knowledge gaps and assessing the clinical benefit: Identifying the knowledge gaps and assessing the clinical benefit. *JAMA Dermatol*. 2020;156(9):1004–11. 32725204 10.1001/jamadermatol.2020.1729 PMC8275355

[10] Multicenter Selective Lymphadenectomy Trials Study Group, Crystal JS, Thompson JF, et al. Therapeutic value of sentinel lymph node biopsy in patients with melanoma: A randomized clinical trial. *JAMA Surg*. 2022;157(9):835–42. 35921122 10.1001/jamasurg.2022.2055 PMC9475390

[11] Agnese DM, Abdessalam SF, Burak WE Jr, Magro CM, Pozderac RV, Walker MJ. Cost-effectiveness of sentinel lymph node biopsy in thin melanomas. *Surgery*. 2003;134(4):542–7 (**discussion 547-8**). 14605613 10.1016/s0039-6060(03)00275-7

[12] Herb JN, Ollila DW, Stitzenberg KB, Meyers MO. Use and costs of sentinel lymph node biopsy in non-ulcerated T1b melanoma: Analysis of a population-based registry. *Ann Surg Oncol*. 2021;28(7):3470–8. 33900501 10.1245/s10434-021-09998-6

[13] Aiken TJ, Stahl CC, Schwartz PB, et al. Sentinel lymph node biopsy is associated with increased cost in higher risk thin melanoma. *J Surg Oncol*. 2021;123(1):104–9. 32939750 10.1002/jso.26225 PMC7770044

[14] Risk of sentinel lymph node metastasis. Memorial Sloan Kettering Cancer Center. https://www.mskcc.org/nomograms/melanoma/sentinel_lymph_node_metastasis. Accessed December 7, 2025.

[15] Olofsson Bagge R, Mikiver R, Marchetti MA, et al. Population-based validation of the MIA and MSKCC tools for predicting sentinel lymph node status. *JAMA Surg*. 2024;159(3):260–8. 38198163 10.1001/jamasurg.2023.6904 PMC10782377

[16] Bartlett EK, O’Donoghue C, Boland G, et al. Society of Surgical Oncology consensus statement: Assessing the evidence for and utility of gene expression profiling of primary cutaneous melanoma. *Ann Surg Oncol*. 2025;32(3):1429–42. 39470890 10.1245/s10434-024-16379-2 PMC11811439

[17] Drebin HM, Hosein S, Kurtansky NR, et al. Clinical utility of melanoma sentinel lymph node biopsy nomograms. *J Am Coll Surg*. 2024;238(1):23–31. 37870230 10.1097/XCS.0000000000000886 PMC11735020

