# Supplementary material for: Patterns and Predictors of Sentinel Lymph Node Biopsy Utilization in High- and Low-Risk Thin Melanomas
Source: Ann Surg Oncol. 2026 Jan 16;33(5):4000–10. doi: 10.1245/s10434-025-19079-7 (PMC13083517; doi:10.1245/s10434-025-19079-7)
Supplement: Supplementary file 1 — Supplementary file1 (DOCX 100 KB) [file 10434_2025_19079_MOESM1_ESM.docx]

**
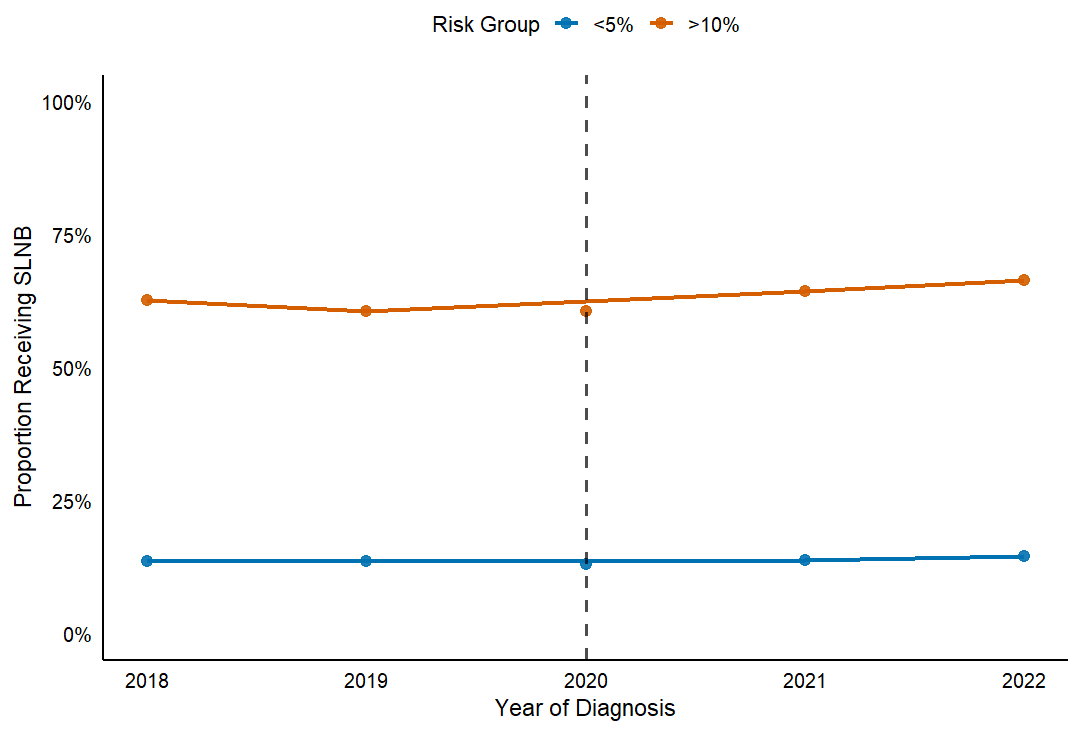
**

**Supplementary Figure.** Trends in sentinel lymph node biopsy (SLNB) utilization over time

before and after the implementation period of the Melanoma Institute of Australia sentinel node

positivity risk tool in 2020.
